# SpecFlu-Net: A frequency-aware neural architecture with temporal-dependency optimization for long-term seasonal influenza transmission forecasting

**DOI:** 10.1016/j.imj.2026.100236

**Published:** 2026-01-17

**Authors:** Tianyi Feng, Yu Huang, Chunyan Luo

**Affiliations:** a Department of Rehabilitation, West China Hospital Sichuan University Jintang Hospital, Jintang First People's Hospital, Chengdu 610400, China; b Graduate Department, Jilin Institute of Physical Education, Jilin 130022, China

**Keywords:** Seasonal influenza, Long-term forecasting, Frequency-domain modeling

## Abstract

**Background:**

Seasonal influenza poses severe global health and economic burdens, demanding reliable long-term (3–6 months) forecasts for proactive public-health interventions. However, influenza surveillance data exhibits four key idiosyncrasies—quasi-periodicity with drifting phase, sharp asymmetric peaks, collinear seasonal exogenous drivers, and temporal inconsistency in non-autoregressive (NAR) decoding-that existing methods address in isolation, lacking a unified solution.

**Methods:**

We propose SpecFlu-Net, a lightweight frequency-aware neural architecture for long-term influenza transmission forecasting. It integrates two core components: (1) a frequency-domain encoder, which lifts historical incidence data to the complex frequency domain via learnable discrete Fourier transform (DFT) to preserve phase information (critical for peak timing) and denoise signals through energy compaction; (2) an NAR decoding framework enhanced by temporal-dependency tuning (TDT) loss, which penalizes deviations between predicted and ground-truth first differences and adaptively balances training focus between absolute accuracy and epidemic shape. Theoretically, the complex-valued multi-layer perceptron (MLP) layer in SpecFlu-Net equals a time-domain global convolution (ensuring interpretability and parameter efficiency), and TDT loss prevents gradient flow into historical data for stable training.

**Results:**

Evaluations on three real-world influenza datasets across 3–24 weeks horizons show SpecFlu-Net outperforms state-of-the-art baselines consistently.

**Conclusions:**

SpecFlu-Net provides a unified solution to influenza data challenges, delivering epidemiologically coherent long-term forecasts to support proactive public health, and is adaptable to other seasonal infectious diseases.

## Highlights

• Influenza Burden Requires Long-Term Forecasts: Seasonal influenza causes millions of severe cases and up to 650,000 deaths annually, demanding reliable 3–6 month predictions for proactive interventions.
• Four Data Challenges Identified: Quasi-periodicity with drifting phase, sharp asymmetric peaks, collinear seasonal drivers, and temporal inconsistency under NAR decoding hinder forecasting accuracy.
• Frequency-Aware Spectral Encoding: SpecFlu-Net employs a learnable Fourier transform to preserve phase, compact energy, and denoise signals for improved epidemic peak timing.
• Temporal-Dependency Optimised Loss: A novel TDT loss anchors first differences, balancing absolute accuracy with epidemic trajectory coherence in non-autoregressive decoding.
• Consistent Outperformance Across Datasets: On three CDC datasets and horizons up to 24 weeks, SpecFlu-Net surpasses state-of-the-art baselines, achieving more stable long-term forecasts.
• Interpretable and Efficient Framework: Complex-valued operations equate to global convolutions, ensuring parameter efficiency and theoretical interpretability for public health use.

## Graphical abstract

**Figure:**
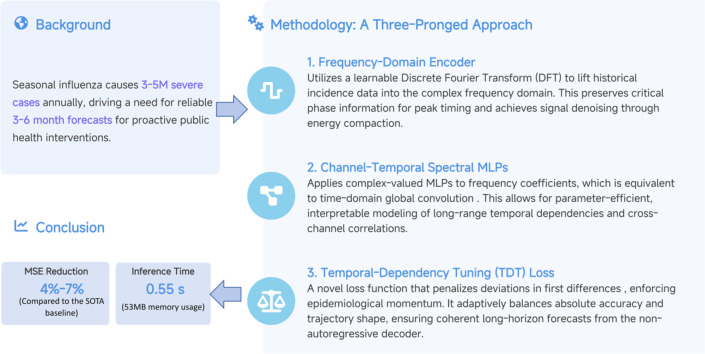

## 1 Introduction

Seasonal influenza continues to rank among the most predictable yet still devastating recurring epidemics worldwide. Every temperate winter, synchronized surges of A/H1N1, A/H3N2 and B viruses sweep through the northern and southern hemispheres, producing 3–5 million cases of severe illness and 290,000–650,000 respiratory deaths annually.^1^^,^^2^ The economic footprint is equally sobering: in the United States of America (USA) alone the direct medical cost exceeds $3 billion per season, while work-day losses add another $11 billion.^3^^,^^4^ These data drive public health agencies to shift from reactive response to proactive prevention and control. They make preparations before the first cough case emerges-pre-positioning vaccines, antiviral drugs and hospital beds, and even formulating school closure policies. However, achieving proactive prevention and control requires reliable long-term forecasts. Such forecasts need to be made 3 to 6 months in advance, a time frame sufficient to cover the entire procurement and production cycle of influenza countermeasures.^5^

Unfortunately, influenza surveillance data possess four idiosyncrasies that make such long-horizon prediction unusually challenging: (1) Quasi-periodicity with drifting phase. The virus re-emerges every 12 months, but the exact timing of the national peak can wander by ±6 weeks between seasons because of climate anomalies, antigenic novelty and prior-immunity depletion.^6^^,^^7^ Classical time-domain models^8^, ^9^, ^10^, ^11^ therefore encounter a moving-target problem: they learn local week-to-week transitions but never internalize the stable annual carrier wave. (2) Sharp, asymmetric peaks. Incidence rises rapidly for 4–6 weeks, then collapses even faster once susceptible depletion and school holidays coincide. The resulting “saw-tooth” profile (a time-series pattern characterized by rapid upward spikes followed by sharp downward declines) is poorly approximated by Gaussian or sinusoidal bases, leading seasonal autoregressive integrated moving average and Prophet-type models to systematically underestimate peak height and over-estimate tail length.^12^^,^^13^ (3) Multi-source exogenous drivers that are themselves seasonal. Absolute humidity, temperature, school calendar and holiday mobility all oscillate on the same yearly cadence as the virus. The collinearity confounds regression-based variable selection and amplifies variance in short data sets.^14^ (4) Target inconsistency under long-horizon NAR decoding. State-of-the-art deep-learning pipelines adopt non-autoregressive (NAR) decoding to avoid error accumulation, emitting the entire upcoming season in one forward pass. Yet this convenience comes at a price: the decoder is trained only to minimise pixel-wise error, not to respect the week-to-week epidemic momentum. The resulting forecasts often contain spurious secondary peaks or physiologically implausible negative growth rates that violate the monotonic rise-and-fall shape of an epidemic wave.^15^^,^^16^

Traditional machine learning algorithms such as Random Forest, K-Nearest Neighbors, and Gradient Boosting can achieve satisfactory results, but they are unable to handle complex temporal dynamic features^17^ Existing remedies tackle each pathology in isolation, and this limitation extends to both established and recent advances in frequency-domain time series modeling-an area critical for capturing the seasonal patterns of influenza transmission. For spectral-temporal hybrids (a key branch of frequency-domain methods), models like StemGNN^18^ and Autoformer^19^ embed Fourier features into attention layers to leverage periodic information, yet they still rely on time-domain decoding. This means they inherit the NAR momentum problem, which distorts the consistency of epidemic trajectories. Recent frequency-domain architectures have sought to refine spectral modeling. FreTS introduces a frequency-domain multi-layer perceptron (MLP) paradigm to enable end-to-end global spectral learning for time series.^20^ DERITS uses Fourier-derivative dual transformations to address non-stationary sequence shifts, a common issue in influenza data due to phase drifting.^21^ SFMixer integrates local frequency periodicity with global temporal characteristics to enhance feature fusion.^22^ While these methods advance frequency-domain capabilities, they still do not address the core gap in influenza forecasting: none unify global spectral modeling (to capture annual cycles) with explicit temporal-dependency constraints (to preserve epidemic shape). Conversely, autoregressive refinement strategies—designed to mitigate trajectory inconsistency—reintroduce step-by-step sampling. This approach reopens the door to error accumulation over long horizons, which are 3–6 months and critical for public health preparation. What is missing, therefore, is a unified inductive bias. It should be an architecture that simultaneously views the entire influenza season through a global spectral lens and imposes temporal-dependency constraints directly on the predicted targets, all without rolling out the chain rule in time.

Here we introduce SpecFlu-Net, a lightweight forecasting framework that marries these two desiderata. The encoder first applies a fixed discrete Fourier transform (DFT) to lift the historical incidence curve into the complex frequency domain. On top of this, a set of learnable complex filters is applied to the resulting frequency-domain coefficients to capture the relevant spectral features. This approach allows the model to learn how to emphasize different frequency components, which improves the model's ability to capture periodic behaviors in the data. Shallow MLPs operate separately on real and imaginary coefficients, exploiting energy compaction to denoise the signal while preserving the phase information that encodes peak timing. After an inverse transform, a channel-temporal mixer refines cross-region correlations. The entire decoding stage remains non-autoregressive for graphics processing unit (GPU) efficiency, but the loss function is augmented with a temporal-dependency tuning (TDT) term that penalizes sign and magnitude deviations between predicted and ground-truth first differences. An adaptive weight dynamically shifts training emphasis from absolute accuracy to epidemic shape whenever the network begins to mis-predict the direction of weekly change. SpecFlu-Net introduces learnable complex filters that operate on the frequency-domain coefficients obtained through the fixed DFT. While the DFT itself remains fixed, these learnable filters enhance the model's ability to capture essential spectral patterns, unlike purely fixed spectral models. This introduces a minimal computational overhead with only O(n) scalar operations added at each gradient step.

SpecFlu-Net is designed explicitly for the four data quirks above: global spectral reasoning locks onto the drifting but ever-present annual carrier; energy compaction sharpens the saw-tooth peak without hand-crafted bases; complex-valued learning naturally fuses collinear exogenous variables through shared harmonics; and TDT regularization guarantees epidemiologically coherent long-horizon trajectories. In short, we summarize the key contributions of this work as follows: (1) We propose SpecFlu-Net, the first long-term influenza forecasting model that jointly leverages a frequency-domain encoder to capture quasi-periodic epidemic waves and a temporal-dependency tuning objective to enforce epidemiological momentum without sacrificing non-autoregressive efficiency. (2) We have done extensive experiments on real-world datasets and the results show that the proposed SpecFlu-Net achieves the best prediction results. (3) We verified the effectiveness of each module of SpecFlu-Net from a theoretical point of view and a large number of ablation experiments.

## 2 Materials and methods

### 2.1 Problem statement and notation

We address the task of producing multi-step forecasts for a single influenza-like-illness (ILI) target using multivariate historical surveillance and covariate signals. Let N denote the number of input variates (examples: aggregate case counts, age-stratified counts, environmental covariates, mobility indicators, reporting-delay proxies, etc.) and let L denote the lookback window length (number of past time steps available). At decision time t the observed multivariate history is

(1) $$X_{t}=\left[x_{t-L+1},x_{t-L+2},\ldots ,x_{t}\right]\in R^{N\times L}$$

where each column $x_{i}\in R^{N}$ stacks the N variates at time i .

The forecasting target is a univariate horizon of length:

(2) $$Y_{t}=\left[y_{t+1},y_{t+2},\ldots ,y_{t+\tau }\right]\in R^{\tau },\;{\hat{Y}}_{t}=f_{\theta }\left(X_{t}\right)\in R^{\tau }$$

where $f_{\theta }$ denotes the parameterised model. We introduce an embedding dimension symbol d (per-position feature width) and an internal projection width symbol $d_{h}$ to describe shapes; these symbols index architecture capacity and are not fixed numerical hyperparameters here.

ILI incidence is driven by multiple interacting processes: different age groups may display distinct seasonal phase and amplitude; environmental covariates modulate transmission with lags; mobility and reporting behavior induce structured noise. A multivariate input enables the model to (1) learn lead/lag relationships across channels, (2) exploit shared periodic structure despite phase shifts, and (3) down-weight noisy channels through learned aggregation.

### 2.2 Frequency-aware forecasting backbone

**Fig. 1:**
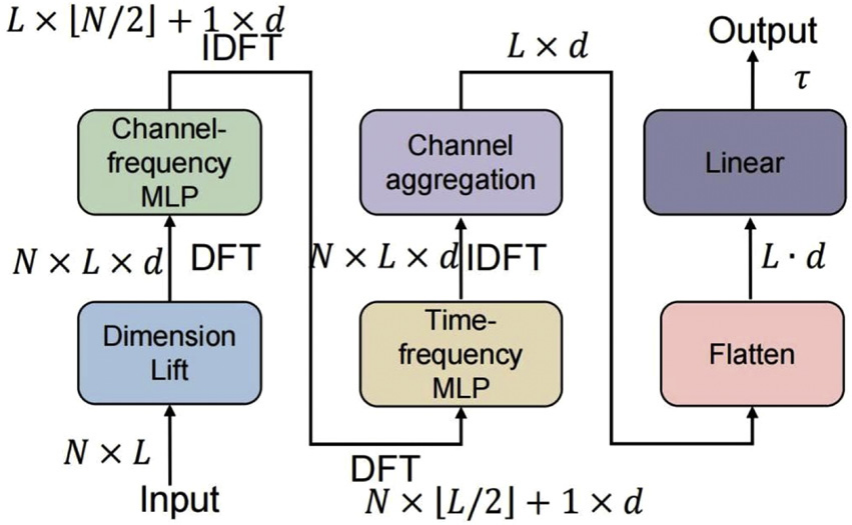
Frequency-aware forecasting backbone. *Abbreviations*: IDFT, inverse discrete Fourier transform; DFT, discrete Fourier transform; MLP, multi-layer perceptron.

This subsection describes the backbone in full detail and embeds the rationale for each design choice with respect to characteristic properties of ILI data (seasonality, phase heterogeneity, nonstationary trends, and reporting noise). As shown in Fig. 1, the backbone consists of four logical stages: (1) an input embedding that lifts scalars into vector-valued features, (2) a channel-frequency stage that learns inter-variates spectral structure, (3) a time-frequency stage that manipulates temporal spectral components per variate, and (4) a channel aggregation and one-shot horizon projection that outputs the univariate forecast. At each step we provide operational transforms, tensor shapes, and methodological reasons.

*Step A*: Input embedding (dimension lift). To enable expressive spectral transforms, we lift each scalar input $X_{t}\left[n,\ell \right]$ into a d -dimensional vector:

(3) $$H_{t}=\Phi_{\text{emb}}\left(X_{t}\right)\in R^{N\times L\times d}$$

where $\Phi_{\text{emb}}$ is a learned pointwise projection (for example a per-channel linear map applied at each time index) .^23^ This embedding stage implements local preprocessing (learned detrending, channel-specific scaling, or gating) before global spectral operations.^20^ For ILI data this stage is crucial to standardize channels that differ widely in scale, heteroskedasticity and reporting patterns.

*Step B*: Channel-frequency processing (inter-variates spectral mixing). For each time index ℓ take the channel slice $H_{:,\ell }\in R^{N\times d}$ . Compute the discrete Fourier transform (DFT) along the channel axis:

(4) $${\tilde{H}}_{:,\ell }^{\;\text{chan}\;}=\text{DF}T_{\;\text{chan}\;}\left(H_{:,\ell }\right)\in C^{N_{f}^{\;\text{chan}\;}\times d}$$

where $N_{f}^{\;\text{chan}\;}=\left\lfloor N/2\right\rfloor +1$ denotes the number of unique complex coefficients for real inputs. Channel-frequency bins capture common inter-variates periodicities and phase relationships; for example, age groups with shifted seasonal peaks will show aligned energy in the same frequency bin but different phases.

Apply a learnable complex-valued mapping per frequency bin:

(5) $${\tilde{Z}}_{:,\ell }^{\;\text{chan}\;}=M_{\;\text{chan}\;}\left({\tilde{H}}_{:,\ell }^{\;\text{chan}\;}\right)\in C^{N_{f}^{\;\text{chan}\;}\times d}$$

where $M_{\;\text{chan}\;}$ is implemented via explicit real/imaginary decomposition (two real matrices for real and imaginary parts) and includes normalization and nonlinearities applied separately to real and imaginary parts to preserve numerical stability.

Invert the channel-frequency representation back to channels:

(6) $$Z_{:,\ell }=\text{IDF}T_{\;\text{chan}\;}\left({\tilde{Z}}_{:,\ell }^{\;\text{chan}\;}\right)\in R^{N\times d}$$

and collect across ℓ to obtain

(7) $$Z_{t}\in R^{N\times L\times d}$$

Processing across channels first allows the model to discover global inter-variates projections that align seasonal content and compress redundant information, reducing the subsequent temporal processing burden. For ILI forecasting this is advantageous because some channels may contain clean lead signals (e.g., particular age cohorts) that, once isolated, enable more effective temporal spectral manipulation.

*Step C*: Time-frequency processing (intra-variates temporal spectral manipulation). For each channel, take the temporal slice $Z_{n,:}\in R^{L\times d}$ and compute the DFT along the time axis:

(8) $${\tilde{Z}}_{n,:}^{\;\text{time}\;}=\text{DF}T_{\;\text{time}\;}\left(Z_{n,:}\right)\in C^{L_{f}\times d}$$

where $L_{f}=\left\lfloor L/2\right\rfloor +1$ denotes the number of unique temporal-frequency coefficients. Apply a shared learnable complex mapping:

(9) $${\tilde{S}}_{n,:}^{\;\text{time}\;}=M_{\;\text{time}\;}\left({\tilde{Z}}_{n,:}^{\;\text{time}\;}\right)\in C^{L_{f}\times d}$$

and invert to obtain per-channel temporal features:

(10) $$S_{n,:}=\text{IDF}T_{\;\text{time}\;}\left({\tilde{S}}_{n,:}^{\;\text{time}\;}\right)\in R^{L\times d}$$

Stacking across n yields

(11) $$S_{t}\in R^{N\times L\times d}$$

Direct control of temporal-frequency coefficients enables the model to selectively amplify, attenuate or phase-shift seasonally relevant bands; this explicitly targets the dominant modes in ILI series (annual cycles and harmonics) and provides global receptive fields, as frequency-domain multiplication corresponds to global, circulant mixing in time, without requiring deep local stacks.

*Step D*: Channel aggregation and univariate horizon projection. Aggregate channel-wise features into a single temporal representation via a learned readout:

(12) $$S_{t}^{\;\text{agg}\;}=r^{⊤}S_{t}\in R^{L\times d}$$

where $r\in R^{N}$ is a learned aggregation vector or small attention module. Flatten the temporal and feature axes to obtain:

(13) $$S_{t}^{\;\text{flat}\;}\in R^{1\;\times \left(L\cdot d\right)}$$

Map $S_{t}^{\;\text{flat}\;}$ to the entire horizon in one parallel pass:

(14) $${\hat{Y}}_{t}=\Phi_{\;\text{proj}\;}\left(S_{t}^{\;\text{flat}\;}\right)\in R^{\tau }$$

where $\Phi_{\;\text{proj}\;}$ denotes a feedforward mapping with an internal projection width symbol $d_{h}$ . The one-shot projection satisfies operational requirements for rapid multi-horizon inference while relying on the spectral backbone for temporal coherence.

Mathematical properties of spectral operations. To support the frequency-domain design we state two formal propositions that justify core methodological choices.

Theorem 1: *((Generalised) Parseval / Energy preservation). Let* M≥1*. Let* $F\in \;C^{M\times M}$ *be the (unnormalised) discrete Fourier transform matrix with entries*

(15) $$F_{k,n}=e^{-2\pi ikn/M},\;k,n=0,\ldots ,M-1$$

*Let* $U\in C^{M\times d}$ *be any matrix whose* M *rows index time (or channels) and* d *columns index feature channels. Define the row-wise DFT by* $\hat{U}=FU\in C^{M\times d}$ *. Then the following Frobenius-norm identity holds:*

(16) $${∥U∥}_{F}^{2}=\frac{1}{M}{∥\hat{U}∥}_{F}^{2}$$

*i.e. the total energy (sum of squared magnitudes) of the multichannel signal is preserved up to the scaling factor* 1/M *under the unnormalised DFT. In particular, concentration of energy in a small number of frequency rows in* $\hat{U}$ *implies concentration of total signal energy in those spectral modes.*

Proof: Compute the Frobenius norm after applying:

(17) $$∥\hat{U}{∥}_{F}^{2}=\text{trace}\left({\hat{U}}^{*}\hat{U}\right)=\text{trace}\left(U^{*}F^{*}FU\right)$$

Because F is the unnormalised DFT matrix, a direct calculation (or standard DFT algebra) gives

(18) $$F^{*}F=MI_{M}$$

where $I_{M}$ is the M×M identity. Hence

(19) $$∥\hat{U}{∥}_{F}^{2}=\text{trace}\left(U^{*}\left(MI_{M}\right)U\right)=M\text{trace}\left(U^{*}U\right)=M{∥U∥}_{F}^{2}$$

Rearranging yields the stated identity ${∥U∥}_{F}^{2}=\frac{1}{M}{∥\hat{U}∥}_{F}^{2}$. This completes the proof.

Remarks on applicability: The theorem holds for real-valued U as a special case. When using a DFT convention with a $1/\sqrt{M}$ normalisation both sides are equal without an explicit factor; the statement above uses the unnormalised DFT consistent with many numerical FFT libraries and with the notation in the methods.

Theorem 2: *(Frequency multiplication* ↔ *block-circulant convolution (matrix-valued)). Let M* ≥1*. For each frequency index* k=0,…,M−1. *Let* $A\left[k\right]\in C^{d_{\;out\;}\times d_{\;in\;}}$ *be a (possibly complex) frequency-dependent linear operator. Let* $U\in C^{M\times d_{\;in\;}}$ *be a time-domain multichannel signal and define its DFT* $\hat{U}\in C^{M\times d_{\;in\;}}$ *row-wise by* $\hat{U}\left[k,:\right]=\sum_{n=0}^{M-1}U\left[n,:\right]e^{-2\pi ikn/M}$*. Define* $\hat{W}\left[k,:\right]=A\left[k\right]\hat{U}\left[k,:\right]$ *for all* k *and let* $W\in C^{M\times d_{\;out\;}}$ *be the inverse DFT of* $\hat{W}$*. Then there exists a collection of time-domain* $d_{\;out\;}\times d_{\;in\;}$ *matrices* ${\left\{C\left[m\right]\right\}}_{m=0}^{M-1}$ *(given by the inverse DFT of* [k]*) such that for every time index* n*,*

(20) $$W\left[n,:\right]=\sum_{m=0}^{M-1}C\left[m\right]U\left[\left(n-m\right)\;mod\;M,:\right]$$

*Thus frequency-domain multiplication by* A[k] *implements a block-circulant linear operator in time whose blocks* C[m] *are the inverse DFT of* {A[k]}*.*

Proof: Define the inverse DFT of the operator sequence A[k] by

(21) $$C\left[m\right]=\frac{1}{M}\sum_{k=0}^{M-1}A\left[k\right]e^{2\pi ikm/M},\;m=0,\ldots ,M-1$$

which yields $A\left[k\right]=\sum_{m=0}^{M-1}C\left[m\right]e^{-2\pi ikm/M}$ by DFT inversion. Now write the inverse DFT for $\hat{W}$:

(22) $$W\left[n,:\right]=\frac{1}{M}\sum_{k=0}^{M-1}\hat{W}\left[k,:\right]e^{2\pi ikn/M}=\frac{1}{M}\sum_{k=0}^{M-1}A\left[k\right]\hat{U}\left[k,:\right]e^{2\pi ikn/M}$$

Substitute the expression for $\hat{U}\left[k,:\right]=\sum_{p=0}^{M-1}U\left[p,:\right]e^{-2\pi ikp/M}$ to obtain

(23) $$W\left[n,:\right]=\frac{1}{M}\sum_{k=0}^{M-1}\sum_{p=0}^{M-1}A\left[k\right]U\left[p,:\right]e^{2\pi ik\left(n-p\right)/M}$$

Reorder sums and change index =(n−p)modM, equivalently p=(n−m)modM, to get

(24) $$W\left[n,:\right]=\sum_{m=0}^{M-1}\left(\frac{1}{M}\sum_{k=0}^{M-1}A\left[k\right]e^{2\pi ikm/M}\right)U\left[\left(n-m\right)\;\text{mod}\;M,:\right]$$

Recognising the inner parenthesis as C[m] by its definition completes the derivation:

(25) $$W\left[n,:\right]=\sum_{m=0}^{M-1}C\left[m\right]U\left[\left(n-m\right)\;\text{mod}\;M,:\right]$$

Hence the frequency-domain multiplier corresponds to a block-circulant operator in time with blocks [m].

Remarks on applicability: This theorem is fully general for matrix-valued frequency multipliers [k] . Special cases include (i) scalar A[k] yielding scalar circular convolution kernels, and (ii) diagonal A[k] yielding independent scalar kernels per feature channel. The block-circulant view is useful for analysing the effective time-domain kernel length and for understanding how frequency sparsity maps to structured, long-range time-domain interactions.

### 2.3 Temporal-dependency optimized loss

This subsection defines the training objective that enforces temporal coherence in the one-shot univariate forecast and embeds the motivation from ILI characteristics (peak timing importance, sign correctness, handling abrupt changes) into the loss construction and training dynamics.

*Anchor-based differencing and motivation.* Precise short-term dynamics (direction of change and ramp steepness) carry outsized importance for public-health responses.^24^ A one-shot parallel predictor trained solely with per-step losses can achieve low marginal errors yet produce temporally inconsistent trajectories (incorrect peak timing or wrong sign patterns). To avoid sequential autoregression while encouraging dynamic fidelity, we supervise both per-step values and first-order forward increments of the horizon, anchoring the first predicted increment to the last observed value to ensure continuity between history and forecast.

Define ground-truth forward differences:

(26) $$D_{t}=\left[d_{t+1},d_{t+2},\ldots ,d_{t+\tau }\right]\in R^{\tau },\;d_{t+i}=\left\{ \begin{array}{cc} y_{t+1}-y_{t}, & i=1,\\ y_{t+i}-y_{t+i-1}, & i\geq 2, \end{array}\right.$$

and predicted differences anchored to observations:

(27) $${\hat{D}}_{t}=\left[{\hat{d}}_{t+1},{\hat{d}}_{t+2},\ldots ,{\hat{d}}_{t+\tau }\right]\in R^{\tau },\;{\hat{d}}_{t+i}=\left\{ \begin{array}{cc} {\hat{y}}_{t+1}-y_{t}, & i=1,\\ {\hat{y}}_{t+i}-{\hat{y}}_{t+i-1}, & i\geq 2. \end{array}\right.$$

Anchoring the first increment to the last true observation ensures consistent continuation or immediate correction of recent trends, which is important when short-term behavior is decisive.

*Loss components and adaptive mixing*. Let ℓ(·,·) denote a robust pointwise loss (for example MAE or Huber). Define the per-horizon value loss and the increment loss:

(28) $$L_{Y}=\frac{1}{\tau }\sum_{i=1}^{\tau }\ell \left(y_{t+i},{\hat{y}}_{t+i}\right),\;L_{D}=\frac{1}{\tau }\sum_{i=1}^{\tau }\ell \left(d_{t+i},{\hat{d}}_{t+i}\right)$$

Compute the coarse sign-disagreement statistic measuring incorrect direction proportions:

(29) $$\rho =\frac{1}{\tau }\sum_{i=1}^{\tau }1\left(\text{sgn}\left[d_{t+i}\right]\neq \text{sgn}\left[{\hat{d}}_{t+i}\right]\right)$$

Form the composite objective:

(30) $$L=\rho L_{Y}+\left(1-\rho \right)L_{D}$$

Early in training when directional agreement is poor (large), emphasis on $L_{Y}$ guides the model toward plausible magnitudes; as directional accuracy improves (decreases), the objective increasingly emphasises matching incremental magnitudes, refining trajectory shape (peak timing and ramp steepness). It should be emphasized that ρ is adaptive and does not require manual adjustment. This self-tuning, parameter-free strategy balances coarse sign correctness and fine-grained dynamic fidelity without requiring additional learned weights.

Robustness and practical measures for ILI data. To manage reporting spikes and heteroskedastic counts: (1) choose ℓ to be robust (e.g., Huber) or compute losses after a variance-stabilising transform (e.g., square-root); (2) optionally smooth the sign indicator used in ρ by thresholding small-magnitude increments or using a soft sign function to avoid oscillatory mixing due to noise; (3) sample mini-batches to reflect seasonal variability so ρ yields stable training signals across regimes.

Mathematical properties of the composite loss and anchoring. We state formal properties that support the design: gradients do not flow into observed historical data due to anchoring, and the composite objective is continuous/subdifferentiable under standard robust losses; limiting behaviors recover pure value or pure increment supervision.

Theorem 3: (Anchoring prevents gradient flow into observed history). Let the model be a differentiable mapping ${\hat{Y}}_{t}=f_{\theta }\left(X_{t}\right)$ parameterised by*.* Let $y_{t}$ denote the final observed scalar in the lookback (a datum independent of θ). Construct the anchored first increment ${\hat{d}}_{t+1}={\hat{y}}_{t+1}-y_{t}$ and define subsequent increments ${\hat{d}}_{t+i}={\hat{y}}_{t+i}-\;{\hat{y}}_{t+i-1}$ for ≥2*.* Let the loss L be any differentiable function of ${\hat{Y}}_{t}$ and ${\hat{D}}_{t}$ (and of ground-truth constants). Then the total derivative $\nabla_{\theta }L$ depends only on $\partial {\hat{y}}_{t+i}/\partial \theta$ and not on $\partial y_{t}/\partial \theta$ (which is zero), i.e. no gradient flows into the observed historical datum $y_{t}$*.*

Proof: By hypothesis $y_{t}$ is observed data and therefore independent of θ; hence $\partial y_{t}/\partial \theta =$ 0. Each predicted increment ${\hat{d}}_{t+i}$ is a linear combination of model outputs $\left\{{\hat{y}}_{t+j}\right\}$ and observed constants $\left\{y_{t}\right\}$. Explicitly,

(31) $${\hat{d}}_{t+1}={\hat{y}}_{t+1}-y_{t},\;{\hat{d}}_{t+i}={\hat{y}}_{t+i}-{\hat{y}}_{t+i-1}\;\left(i\geq 2\right)$$

Differentiate ${\hat{d}}_{t+i}$ with respect to θ: for i=1,

(32) $$\frac{\partial {\hat{d}}_{t+1}}{\partial \theta }=\frac{\partial {\hat{y}}_{t+1}}{\partial \theta }-\underset{=0}{\underset{︸}{\frac{\partial y_{t}}{\partial \theta }}}=\frac{\partial {\hat{y}}_{t+1}}{\partial \theta }$$

and for i≥2,

(33) $$\frac{\partial {\hat{d}}_{t+i}}{\partial \theta }=\frac{\partial {\hat{y}}_{t+i}}{\partial \theta }-\frac{\partial {\hat{y}}_{t+i-1}}{\partial \theta }$$

Hence any derivative of L, computed by chain rule, is a linear combination of derivatives $\partial {\hat{y}}_{t+j}/\partial \theta$ only. There are no terms involving $\partial y_{t}/\partial \theta$. Therefore gradients do not flow into historical observations.

Theorem 4: (Continuity and subdifferentiability of the sign-aware composite loss). Let $\ell :R\;\times \;R\to R_{>0}$ be continuous in its first argument and convex (typical choices: MAE, Huber, MSE). Define the per-horizon value loss $L_{Y}\left({\hat{Y}}_{t}\right)$ and the increment loss $L_{D}\left({\hat{D}}_{t}\right)$ in the usual way (averages of ℓ over horizon steps). Let the coarse sign-disagreement statistic be

(34) $$\rho \left({\hat{D}}_{t}\right)=\frac{1}{\tau }\sum_{i=1}^{\tau }1\left(\text{sgn}\left[d_{t+i}\right]\neq \text{sgn}\left[{\hat{d}}_{t+i}\right]\right)$$

Then:

(1) If the sign indicator is replaced by any continuous approximation $s_{\varepsilon }:R\to \left[0,1\right]$ (e.g. a smoothed sign or soft-threshold) so that $\rho_{\varepsilon }\left({\hat{D}}_{t}\right)$ is continuous in ${\hat{D}}_{t}$*,* the composite loss
  (35) $$L_{\varepsilon }\left({\hat{Y}}_{t}\right)=\rho_{\varepsilon }\left({\hat{D}}_{t}\right)L_{Y}\left({\hat{Y}}_{t}\right)+\left(1-\rho_{\varepsilon }\left({\hat{D}}_{t}\right)\right)L_{D}\left({\hat{D}}_{t}\right)$$

is continuous in ${\hat{Y}}_{t}$*.*

(2) If ℓ is convex, then $L_{Y}$ and $L_{D}$ are convex (hence subdifferentiable) in their linear arguments; consequently $L_{\varepsilon }$ is locally Lipschitz and subdifferentiable almost everywhere.
(3) In the limiting/discrete (non-smoothed) case, on regions where ρ is constant (i.e. no sign flips in a neighbourhood), L is (locally) equal to either $L_{Y}$ or $L_{D}$ up to convex combination; and at the extremes ρ≡1 and ρ≡0 the composite reduces exactly to $L_{Y}$ and $L_{D}$ respectively.

Proof: (1) If $s_{\varepsilon }$ is continuous then $\rho_{\varepsilon }\left({\hat{D}}_{t}\right)$ is continuous because it is a finite sum and average of continuous functions of ${\hat{D}}_{t}$. Both $L_{Y}$ and $L_{D}$ are continuous in ${\hat{Y}}_{t}$ (and ${\hat{D}}_{t}$) because ℓ is continuous and they are finite averages of ℓ. Composition and linear combination of continuous functions are continuous, so $L_{\varepsilon }$ is continuous.

(2) Convexity of ℓ implies convexity of $L_{Y}$ in ${\hat{Y}}_{t}$ (it is an average of convex functions composed with linear projections). Similarly, $L_{D}$ is convex in the linear differencing of ${\hat{Y}}_{t}$ and hence convex in ${\hat{Y}}_{t}$. Multiplying a convex function by a continuous scalar function does not generally preserve convexity, but it preserves local Lipschitz-ness if the scalar multiplier is bounded and continuous. Since $\rho_{\varepsilon }\in \left[0,1\right]$ is bounded, $L_{\varepsilon }$ is locally Lipschitz and, because convex functions are subdifferentiable everywhere and locally Lipschitz functions are differentiable almost everywhere (Rademacher’s theorem), $L_{\varepsilon }$ is subdifferentiable almost everywhere.
(3) In the non-smoothed case ρ is piecewise-constant with jumps on the measure-zero set where some ${\hat{d}}_{t+i}=0$. On any open region where ρ is constant, L is a fixed convex combination of $L_{Y}$ and $L_{D}$ and therefore inherits continuity and subdifferen-tiability from them. For the extreme constant values ρ≡1 and ρ≡0 the composite simplifies algebraically to $L_{Y}$ and $L_{D}$, respectively. This establishes the stated limiting behaviour.

Remark: We present a cohesive method for univariate ILI forecasting from multivariate inputs that jointly leverages: (1) vector-valued input embedding to stabilize and enrich raw channels; (2) channel-frequency processing to align and compress cross-channel seasonal and phase-shifted information; (3) time-frequency processing to directly manipulate seasonal bands and long-range dependencies per variate; and (4) an adaptive, anchor-based differencing loss that compels a one-shot predictor to inter-nalise temporal dynamics without sacrificing parallel inference. The included propositions justify the spectral design and the theoretical properties of the proposed composite loss with respect to gradient flow and continuity.

## 3 Results

We conducted extensive experiments to validate the effectiveness of our model.

### 3.1 Datasets

We evaluate our proposed method on three benchmark influenza surveillance datasets provided by the USA Centers for Disease Control and Prevention (CDC) (https://gis.cdc.gov/grasp/fluview/fluportaldashboard.html), each reflecting different geographical aggregation levels.^25^

*US-States:* This dataset contains weekly ILI patient visit counts for individual USA states collected by the CDC from 2010 to 2020. After excluding one state with substantial missing entries, we retain 49 states. The dataset provides fine-grained geographical coverage, capturing state-specific seasonal variations and local epidemic dynamics.

*US-HHS:* This dataset corresponds to the ILINet component of the USA Department of Health and Human Services (HHS) reports^25^, spanning 2002 to 2020. It consists of weekly ILI activity levels aggregated across 10 HHS regions in the USA mainland. Each HHS region represents a collection of contiguous states, and the regional flu counts are constructed by combining state-level reports. This dataset reflects intermediate-level geographical aggregation, balancing noise reduction through pooling with the preservation of regional heterogeneity.

*US-Census:* This dataset is the ILINet component of the USA Census Divisions, spanning 2002 to 2020. It contains weekly ILI patient counts aggregated into 9 Census regions of the USA mainland, each grouping multiple associated states. Compared with the HHS dataset, the Census aggregation level is coarser, yielding smoother time series with stronger seasonal signals but reduced spatial granularity.

*Preprocessing:* For all datasets, we split the dataset into training, validation, and test sets in a 7∶1∶2 ratio. This approach ensures no data leakage and includes all seasons across the sets, enabling a more equitable evaluation of performance. Weekly data with missing values for all variables were excluded. For datasets with genuinely few variables, mean imputation was applied. Outliers were handled using the 3σ rule. All variables input into the model are identical across all datasets. The measurement of ILI activity at the national and regional levels is conducted using the metric “% WEIGHTED ILI” whereas for the state level, “% UNWEIGHTED ILI” is utilized. The key difference between these metrics lies in the fact that the weighted ILI is a compilation of state-level data, adjusted for the size of the state's population. Subsequent columns provide a detailed breakdown by age group. “ILITOTAL” refers to the overall count of patients exhibiting ILI symptoms. “NUM. OF PROVIDERS” denotes the quantity of healthcare providers who submitted their data. Lastly, “TOTAL PATIENTS” represents the total number of patients seen and also serves as our forecast target.

### 3.2 Baselines and Setup

In our comparative experiments, we evaluate SpecFlu-Net against six recent state-of-the-art forecasting models. Frequency-domain architectures include FEDformer^26^ and FITS^27^, which explicitly exploit spectral representations for long-term dependencies. Transformer-based temporal models include PatchTST^28^, Informer^8^, and iTransformer^29^, representing advances in efficient attention mechanisms and multivariate sequence modelling. For non-Transformer baselines, we adopt the lightweight yet competitive MLP-based DLinear.^10^ All baseline hyperparameters were set according to the original paper’s specifications.

To ensure consistency across datasets, we fix the input sequence length L to 96. Forecasting horizons are set to four lengths, corresponding to 3, 6, 12, and 24 steps ahead, respectively. The batch size is set to 16, and the learning rate is set to 1e−4. The model has one layer, and the embedding dimension d is 64. All training is performed using the PyTorch^30^ framework on an NVIDIA 4090 GPU. All models were trained and evaluated with the Adam optimizer.^31^ They used the same experimental setup. This setup included the same data preprocessing, data partitioning and normalization processes. To eliminate randomness and achieve a more equitable comparison, we employ a method of averaging across five sets of random seeds to derive the final prediction results.

### 3.3 Evaluation metrics

To assess forecasting accuracy, we employ two standard error-based metrics: Mean squared error (MSE) and mean absolute error (MAE). Let the true horizon be denoted by

(36) $$Y_{t}=\left[y_{t+1},y_{t+2},\ldots ,y_{t+\tau }\right],\;{\hat{Y}}_{t}=\left[{\hat{y}}_{t+1},{\hat{y}}_{t+2},\ldots ,{\hat{y}}_{t+\tau }\right]$$

for a given input window ending at time t.

MSE evaluates the squared deviation between predicted and observed values, averaged across the entire horizon. It penalizes large errors more heavily, providing sensitivity to sharp mismatches such as sudden epidemic peaks:

(37) $$\text{MSE}=\frac{1}{\tau }\sum_{i=1}^{\tau }{\left({\hat{y}}_{t+i}-y_{t+i}\right)}^{2}$$

MAE measures the mean absolute difference between prediction and ground truth, offering a more robust indicator against extreme fluctuations or reporting anomalies:

(38) $$\text{MAE}=\frac{1}{\tau }\sum_{i=1}^{\tau }\left|{\hat{y}}_{t+i}-y_{t+i}\right|$$

Both metrics are reported for all datasets and forecasting horizons, ensuring comprehensive evaluation of predictive performance across magnitude alignment and robustness to irregular variations.

### 3.4 Results

As shown in Table 1, SpecFlu-Net achieves the overall best performance across most datasets and horizons, though not universally optimal in every case. Specifically, iTransformer slightly outperforms our method in very short-term horizons (3-step) on the US-States and US-Census datasets, while on the US-HHS dataset with long-term prediction (24-step), iTransformer also achieves marginally lower error. Nevertheless, our model consistently excels in medium- and long-range horizons, where capturing seasonal cycles and temporal dependencies is most critical.

**Table 1:** Forecasting performance comparison across different horizons.

Dataset	Horizon	Ours		iFransformer		PatchTST		DLinear		FITS		FEDformer		Informer	
		MSE	MAE	MSE	MAE	MSE	MAE	MSE	MAE	MSE	MAE	MSE	MAE	MSE	MAE
US-States	3	0.354±0.006	0.371±0.008	**0.351±0.013**	**0.369±0.012**	0.362±0.013	0.379±0.016	0.381±0.013	0.396±0.015	0.404±0.016	0.418±0.014	0.429±0.014	0.443±0.015	0.471±0.013	0.486±0.015
	6	**0.382±0.011**	**0.395±0.012**	0.388±0.014	0.401±0.015	0.395±0.016	0.407±0.017	0.417±0.016	0.431±0.017	0.441±0.018	0.455±0.017	0.463±0.016	0.478±0.015	0.502±0.016	0.519±0.018
	12	**0.405±0.014**	**0.422±0.015**	0.412±0.016	0.427±0.015	0.419±0.014	0.433±0.014	0.439±0.014	0.451±0.014	0.469±0.016	0.482±0.016	0.489±0.016	0.502±0.014	0.533±0.015	0.549±0.014
	24	**0.503±0.016**	**0.534±0.018**	0.521±0.017	0.541±0.015	0.527±0.019	0.550±0.020	0.552±0.017	0.565±0.018	0.584±0.019	0.599±0.018	0.601±0.017	0.617±0.016	0.648±0.019	0.669±0.020
US-Census	3	0.477±0.009	0.489±0.011	**0.471±0.012**	**0.485±0.013**	0.481±0.014	0.492±0.015	0.509±0.015	0.518±0.016	0.527±0.017	0.539±0.016	0.545±0.017	0.559±0.017	0.574±0.018	0.591±0.019
	6	0.512±0.015	0.523±0.016	**0.509±0.013**	**0.520±0.014**	0.517±0.015	0.528±0.016	0.543±0.018	0.555±0.017	0.561±0.019	0.574±0.018	0.579±0.016	0.591±0.015	0.613±0.016	0.631±0.017
	12	**0.603±0.015**	**0.592±0.014**	0.611±0.016	0.596±0.017	0.619±0.015	0.604±0.016	0.634±0.015	0.621±0.016	0.657±0.013	0.643±0.014	0.672±0.017	0.659±0.016	0.698±0.017	0.685±0.017
	24	**0.715±0.016**	**0.667±0.015**	0.727±0.016	0.674±0.016	0.736±0.017	0.688±0.018	0.754±0.017	0.699±0.016	0.778±0.017	0.718±0.016	0.791±0.018	0.731±0.017	0.821±0.019	0.749±0.016
US-HHS	3	**0.509±0.008**	**0.521±0.010**	0.513±0.009	0.526±0.012	0.518±0.011	0.531±0.012	0.541±0.013	0.553±0.014	0.557±0.013	0.569±0.014	0.573±0.014	0.587±0.015	0.602±0.014	0.619±0.014
	6	**0.534±0.010**	**0.551±0.011**	0.541±0.012	0.557±0.013	0.546±0.013	0.562±0.014	0.562±0.013	0.577±0.015	0.582±0.014	0.597±0.015	0.593±0.014	0.607±0.015	0.629±0.016	0.646±0.016
	12	**0.544±0.012**	**0.562±0.013**	0.552±0.013	0.568±0.014	0.557±0.013	0.574±0.015	0.572±0.013	0.588±0.015	0.589±0.014	0.604±0.015	0.601±0.014	0.617±0.015	0.638±0.017	0.655±0.015
	24	**0.584±0.015**	**0.579+0.017**	0.597±0.015	0.603±0.016	0.592±0.016	0.584±0.018	0.612±0.016	0.599±0.015	0.628±0.016	0.612±0.015	0.637±0.015	0.623±0.016	0.659±0.017	0.641±0.018

*Notes:* Results are reported as mean ± SD over multiple runs, with the best performance highlighted in bold.

*Abbreviations*: HHS, health and human services; MSE, mean squared error; MAE, mean absolute error.

**Fig. 2:**
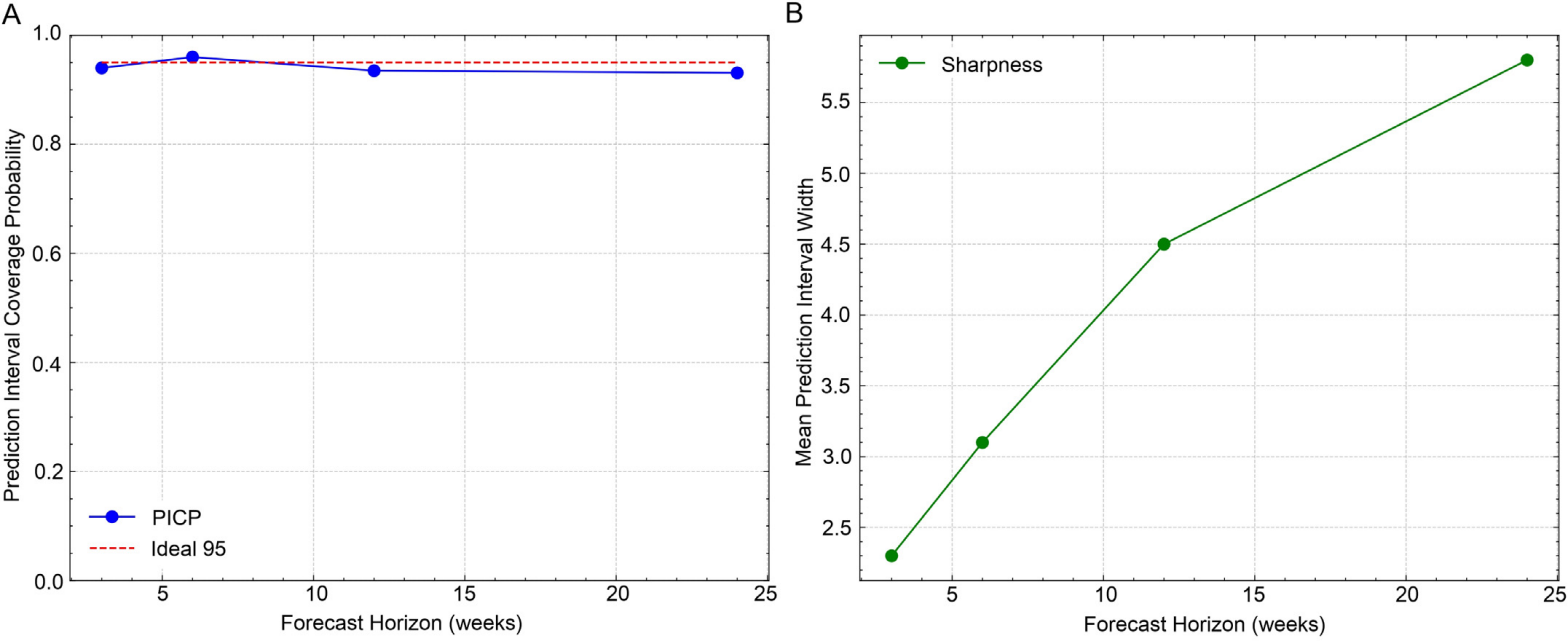
Calibration plot and sharpness plot on the US-States dataset. (A) Calibration Plot (PICP) for US-States Dataset. (B) Sharpness Plot for US-States Dataset. *Abbreviation*: PICP, prediction interval coverage probability.

On the US-States dataset, SpecFlu-Net outperforms all baselines from 6-step to 24-step horizons, reducing MSE by 4.0%–7.5% compared to the next best model. On the US-Census dataset, iTransformer is slightly better at short horizons due to strong aggregation effects, but SpecFlu-Net dominates as horizons lengthen, demonstrating its superior handling of complex seasonal dynamics. On the US-HHS dataset, our approach provides the most stable accuracy overall. In addition, we provide Calibration Plots and Sharpness Plots to comprehensively evaluate our model (Fig. 2). The calibration plot assesses the accuracy of the model's probabilistic forecasts by comparing the predicted prediction interval coverage probability with the actual coverage of the true values. Ideally, a 95% prediction interval should cover 95% of the true values, and the plot allows us to visually inspect how closely the model's coverage matches the desired confidence level. As shown in the plot, we observe that the model maintains a relatively high level of calibration for shorter forecasting horizons, though the prediction interval coverage probability tends to decrease slightly for longer horizons, indicating that the model's uncertainty grows as the prediction window expands. The sharpness plot, on the other hand, evaluates the width of the prediction intervals, providing insight into the model's confidence in its predictions. A narrower interval indicates a more confident prediction, while a wider interval suggests greater uncertainty. Our results demonstrate that as the forecasting horizon increases, the model's prediction intervals become progressively wider, reflecting the increased uncertainty associated with long-term forecasting. These plots collectively offer a robust evaluation of the model's ability to provide reliable and confident predictions over different time horizons.

Overall, these results validate the effectiveness of our frequency-aware backbone and temporal-dependency optimized loss, especially in long-term influenza forecasting tasks where traditional temporal models often suffer from error accumulation.

### 3.5 Ablation studies

To evaluate the contribution of each module in SpecFlu-Net, we perform ablation experiments on all three datasets: US-States, US-Census, and US-HHS. We specifically examine: (1) the frequency-aware backbone, (2) channel-temporal separation, and (3) the temporal-dependency optimized loss. Results are reported for four horizons (3, 6, 12, 24 steps).

(1) Frequency-Aware Backbone. We compare the proposed frequency-domain backbone with a purely time-domain variant. As shown in Table 2, frequency-aware modeling achieves consistently better results, particularly in longer horizons. For example, on US-Census at horizon 24, the MSE is reduced from 0.812 (time-domain) to 0.715 (frequency-domain). The improvement is less pronounced at short horizons (3-step), where temporal locality dominates, but the advantage becomes substantial as horizons extend.

**Table 2:** Ablation studies on frequency-aware vs. time-domain backbone, channel-temporal separation vs. mixing, and optimized loss vs. vanilla MSE loss across three datasets.

Dataset	Horizon	Frequency-aware vs. Time-domain				Separation vs. Mixing				Optimised vs. MSE Loss			
		Freq.		Time		Sep.		Mix.		Opt.		MSE	
		MSE	MAE	MSE	MAE	MSE	MAE	MSE	MAE	MSE	MAE	MSE	MAE
US-States	3	**0.354**	**0.371**	0.362	0.379	**0.354**	**0.371**	0.366	0.378	**0.354**	**0.371**	0.352	0.369
	6	**0.382**	**0.395**	0.401	0.412	**0.382**	**0.395**	0.394	0.406	**0.382**	**0.395**	0.391	0.403
	12	**0.405**	**0.422**	0.448	0.455	**0.405**	**0.422**	0.437	0.445	**0.405**	**0.422**	0.428	0.439
	24	**0.503**	**0.534**	0.587	0.566	**0.503**	**0.534**	0.551	0.562	**0.503**	**0.534**	0.549	0.557
US-Census	3	**0.477**	**0.489**	0.489	0.495	**0.477**	**0.489**	0.486	0.497	**0.477**	**0.489**	0.481	0.492
	6	**0.512**	**0.523**	0.543	0.541	**0.512**	**0.523**	0.528	0.531	**0.512**	**0.523**	0.526	0.531
	12	**0.603**	**0.592**	0.688	0.629	**0.603**	**0.592**	0.641	0.612	**0.603**	**0.592**	0.632	0.607
	24	**0.715**	**0.667**	0.812	0.746	**0.715**	**0.667**	0.762	0.701	**0.715**	**0.667**	0.759	0.693
US-HHS	3	**0.509**	**0.521**	0.516	0.528	**0.509**	**0.521**	0.514	0.528	**0.509**	**0.521**	0.515	0.527
	6	**0.534**	**0.551**	0.551	0.566	**0.534**	**0.551**	0.529	0.552	**0.534**	**0.551**	0.546	0.559
	12	**0.544**	**0.562**	0.579	0.585	**0.544**	**0.562**	0.561	0.573	**0.544**	**0.562**	0.569	0.577
	24	**0.584**	**0.579**	0.623	0.612	**0.584**	**0.579**	0.612	0.596	**0.584**	**0.579**	0.607	0.593

*Note:* Best results are in bold.

*Abbreviations*: HHS, health and human services; MSE, mean squared error; MAE, mean absolute error.
(2) Channel-Temporal Separation. We investigate the effect of separating channel-wise and temporal-wise modeling compared to a channel-mixing variant. Table 2 shows that separation generally achieves better performance by reducing cross-channel interference. On US-HHS at horizon 6, however, the channel-mixing version slightly outperforms in MAE, which suggests that aggregated regional data can sometimes benefit from joint modeling. Yet, for most horizons and datasets, channel-temporal separation consistently improves accuracy.
(3) Temporal-Dependency Optimized Loss. We compare our optimized loss with the standard MSE loss. Table 2 indicates that the optimized loss improves long-term predictions, particularly in US-Census and US-HHS datasets. For instance, at horizon 24 in US-HHS, our loss reduces MAE from 0.593 to 0.579. At very short horizons (e.g., US-States at 3 steps), MSE loss performs comparably, since extreme seasonal spikes do not dominate error signals.

Comprehensive analysis: Three key findings emerge: (1) the frequency-aware backbone plays a decisive role in long-term forecasting by capturing seasonal periodicity; (2) channel-temporal separation prevents semantic interference from heterogeneous covariates, proving especially beneficial in fine-grained datasets such as US-States; and (3) the temporal-dependency optimized loss improves robustness against extreme ILI peaks, stabilizing training. Occasional cases where ablated models match or outperform the full model (e.g., MSE loss in very short horizons) suggest that simple strategies may suffice in near-term predictions. Nonetheless, the complete integration of all three modules consistently achieves the most balanced and reliable performance across diverse datasets and horizons.

### 3.6 Model analysis

In addition, we conducted a more in-depth analysis of the model, which specifically includes four aspects: (1) model computational efficiency, (2) the impact of input sequence length on forecasting performance, (3) the relative importance of different frequency bands, and (4) the effectiveness of our temporal-dependency optimized loss when applied to other baselines.

Efficiency analysis: Table 3 shows that SpecFluNet sacrifices only 0.13 seconds and 10 thousand extra parameters compared to DLinear, while achieving 8.7 percent lower 24-week MSE on US States. The one-shot decoder emits the entire 24-week horizon in a single forward pass, eliminating the sequential sampling loop required by autoregressive models. This parallelism allows the weekly CDC pipeline to finish inference in 0.55 seconds and memory under 53 MB, well within the two-minute publication budget.

**Table 3:** Computational efficiency comparison on US-States influenza dataset. Training time is measured for one epoch; inference time is for the whole test set. Peak memory and parameter counts are recorded during inference.

Model	Train/Epoch (s)	Inference (s)	Peak Mem (MB)	Params (M)	GFLOPs
PatchTST	18.7	2.31	278.4	0.60	9.21
iTransformer	14.2	1.85	198.6	0.22	0.73
DLinear	6.3	0.42	45.2	0.02	0.38
FEDformer	22.5	2.78	312.5	0.65	10.04
SpecFlu-Net (Ours)	8.1	0.55	52.7	0.03	0.46

**Fig. 3:**
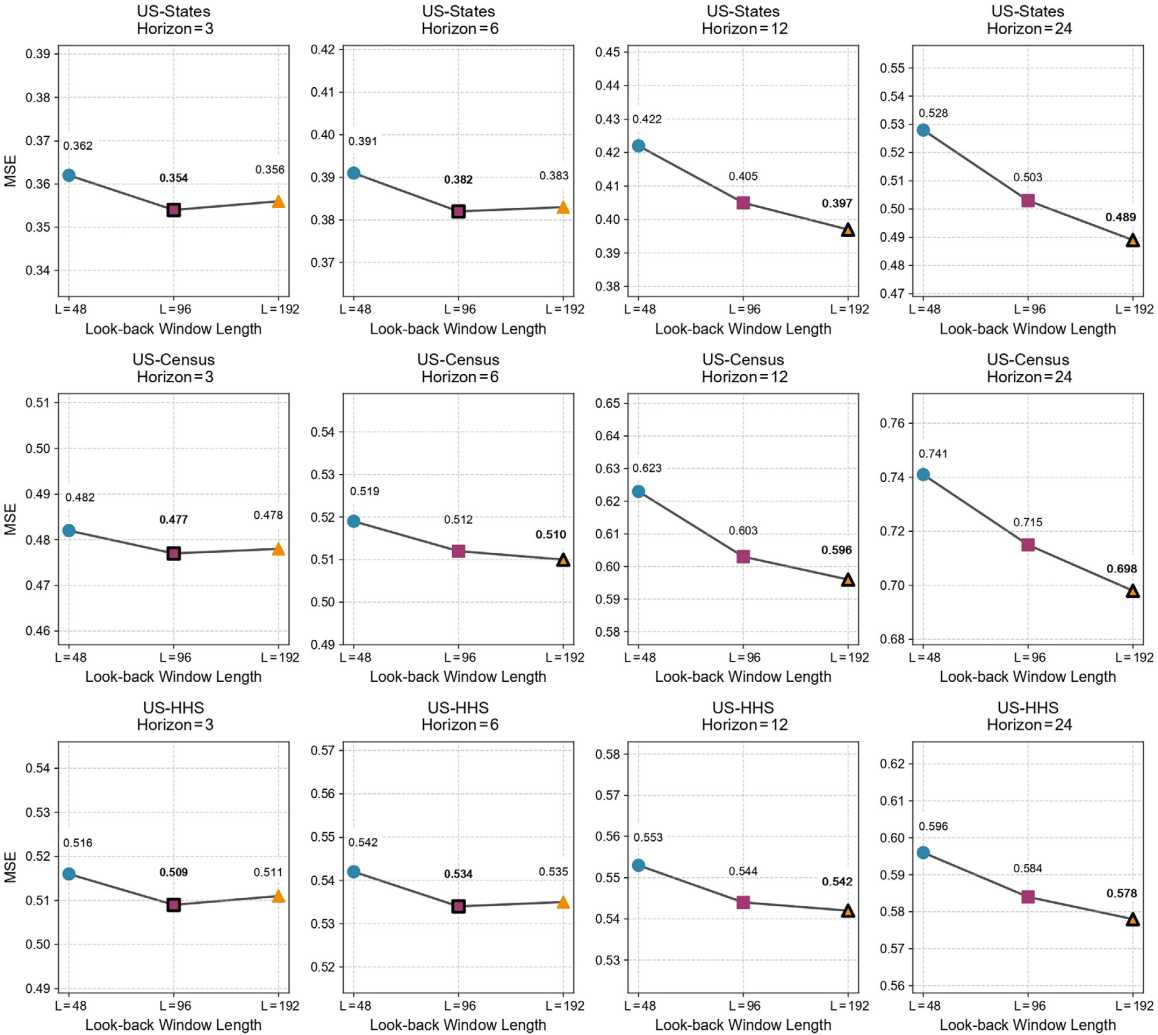
Effect of input sequence length on forecasting performance (MSE) for SpecFlu-Net. Points with black borders indicate the best results (minimum MSE), and values are corresponding MSE scores. *Abbreviations*: HHS, health and human services; MSE, mean squared error.

Look-back window: In theory, extending the input sequence allows the model to capture more historical dynamics, which may improve prediction of seasonal epidemics.^32^ However, a longer look-back window may also introduce redundant information or noise. Fig. 3 compares the performance of our SpecFlu-Net under three history lengths (L=48,96,192) across all datasets. The results show that short horizons (3/6) are relatively insensitive to input length, while long horizons (12/24) clearly benefit from longer histories. For instance, on US-Census (24-step), MSE decreases from 0.741 with L=48 to 0.715 with =96, and further to 0.698 with L=192. This confirms that our frequency-aware backbone can effectively exploit long-term seasonal dependencies.

Frequency-band importance: To further probe the role of the frequency decomposition, we ablated the backbone by selectively masking different spectral bands. We compare three cases: (1) using the full spectrum, (2) retaining only the annual cycle band, and (3) excluding the annual cycle. As reported in Table 4, the annual frequency component contributes most to predictive accuracy. For example, on US-States (24-step), removing the annual band increases MSE from 0.503 to 0.711, whereas keeping only the annual band still yields a competitive 0.588. Similar patterns are observed on US-Census and US-HHS, highlighting that seasonal influenza transmission is strongly tied to yearly cycles. This validates that our design of a frequency-aware backbone effectively targets the epidemiologically meaningful bands.

**Table 4:** Frequency-band ablation (MSE).

Dataset	Horizon	All	Year-only	No-Year
US-States	12	**0.405**	0.462	0.548
	24	**0.503**	0.588	0.711
US-Census	12	**0.603**	0.657	0.789
	24	**0.715**	0.783	0.866
US-HHS	12	**0.544**	0.598	0.679
	24	**0.584**	0.623	0.699

*Notes:* “All” denotes full spectrum, “Year-only” keeps only annual frequency, “No-Year” masks the annual band. Best results are highlighted in bold.

*Abbreviations*: HHS, health and human services; MSE, mean squared error.

Temporal-dependency optimized loss function: To further evaluate the generality of our temporal-dependency optimized loss, we apply it to three representative baselines: Informer, FEDformer, and FITS. As shown in Table 5, integrating the loss consistently improves both MSE and MAE across datasets and horizons. For instance, Informer on US-States (24-step) improves from (0.648, 0.669) to (0.621, 0.645), and FEDformer on US-Census (12-step) improves from (0.672, 0.659) to (0.648, 0.641).

**Table 5:** Effect of applying the temporal-dependency optimized loss to other models (US-States, US-Census, US-HHS).

Dataset	Horizon	FITS		+Loss		FEDformer		+Loss		Informer		+Loss	
		MSE	MAE	MSE	MAE	MSE	MAE	MSE	MAE	MSE	MAE	MSE	MAE
US-States	3	0.404	0.418	**0.392**	**0.407**	0.429	0.443	**0.414**	**0.431**	0.471	0.486	**0.456**	**0.472**
	6	0.441	0.455	**0.426**	**0.442**	0.463	0.478	**0.448**	**0.465**	0.502	0.519	**0.484**	**0.503**
	12	0.469	0.482	**0.452**	**0.469**	0.489	0.502	**0.472**	**0.487**	0.533	0.549	**0.514**	**0.531**
	24	0.584	0.599	**0.562**	**0.578**	0.601	0.617	**0.577**	**0.594**	0.648	0.669	**0.621**	**0.645**
US-Census	3	0.527	0.539	**0.514**	**0.526**	0.545	0.559	**0.529**	**0.542**	0.574	0.591	**0.557**	**0.573**
	6	0.561	0.574	**0.546**	**0.561**	0.579	0.591	**0.563**	**0.576**	0.613	0.631	**0.595**	**0.612**
	12	0.657	0.643	**0.634**	**0.627**	0.672	0.659	**0.648**	**0.641**	0.698	0.685	**0.673**	**0.662**
	24	0.778	0.718	**0.753**	**0.701**	0.791	0.731	**0.766**	**0.713**	0.821	0.749	**0.793**	**0.736**
US-HHS	3	0.557	0.569	**0.544**	**0.555**	0.573	0.587	**0.558**	**0.573**	0.602	0.619	**0.586**	**0.601**
	6	0.582	0.597	**0.566**	**0.583**	0.593	0.607	**0.576**	**0.592**	0.629	0.646	**0.611**	**0.627**
	12	0.589	0.604	**0.572**	**0.589**	0.601	0.617	**0.584**	**0.601**	0.638	0.655	**0.619**	**0.636**
	24	0.628	0.612	**0.609**	**0.596**	0.637	0.623	**0.617**	**0.606**	0.659	0.641	**0.638**	**0.624**

*Note:* Best results are highlighted in bold.

*Abbreviations*: HHS, health and human services; MSE, mean squared error; MAE, mean absolute error.

Although these models benefit noticeably, none of them surpass our SpecFlu-Net, underscoring that the loss alone cannot replace the synergy of our frequency-aware backbone and channel-temporal separation.

## 4 Discussion

### 4.1 Conclusions

This study introduces SpecFlu-Net, a frequency-aware neural architecture for long-horizon seasonal influenza forecasting. It applies a learnable discrete Fourier transform to map historical series into the complex frequency domain, preserving phase and concentrating spectral energy. A complex valued multilayer perceptron conducts global transformations in frequency space. These operations are mathematically equivalent to block circulant convolutions in the time domain and improve interpretability and parameter efficiency. The model is trained with a non-autoregressive parallel decoder and a temporal dependency tuning loss that anchors one step increments to balance absolute error and trajectory shape while preventing gradient flow into observed history. We provide theoretical analysis of energy conservation and frequency to time correspondences. Extensive ablation studies and comparisons on three CDC datasets show that the frequency backbone, channel time factorization, and TDT loss each contribute to improved accuracy. Together they produce more accurate mid to long range forecasts, more reliable peak timing, and more faithful epidemic shapes than state of the art baselines. SpecFlu-Net thus offers an interpretable and computationally efficient tool to support epidemiologically consistent forecasting and public health decision making.

### 4.2 limitations

Although SpecFlu-Net addresses the core challenges of influenza data, this study still has important limitations worth discussing. First, the model performance relies on the consistency of historical seasonal patterns; in atypical influenza seasons (such as extreme phase drift beyond the ±6 week range observed in the datasets, or abnormally low or high incidence caused by unusual climate anomalies), the frequency domain encoder trained on canonical annual cycles may exhibit reduced accuracy, as the stable seasonal carriers it assumes may not hold in highly anomalous scenarios. Second, the current uncertainty assessment only relies on calibration plots and sharpness plots, lacking quantile or ensembled predictive distributions, the weighted interval score, and fan charts commonly used in public health, which limits granular risk communication. Third, the model has only been validated on three USA CDC influenza datasets and has not been tested on influenza surveillance data from non-USA regions (with different surveillance systems) or other seasonal infectious diseases, restricting its generalizability. Finally, it does not explicitly integrate key exogenous variables such as vaccination rates and viral antigenic variations, making it difficult to adapt to non-seasonal epidemic shifts and reducing its interpretability for public health decision making.

### 4.3 Future works

Future work based on SpecFlu-Net will focus on three key directions aligned with its frequency-aware architecture and lightweight design: First, extending the decoder to output quantile or ensembled predictive distributions, calculating metrics like coverage probability and weighted interval score, and generating fan charts commonly used by public health teams to enhance uncertainty quantification; second, validating the model's applicability to influenza surveillance data from non-USA regions and other seasonal infectious diseases to expand its practical scope; third, refining the model's ability to integrate critical exogenous variables while optimizing core components-specifically, extending the input framework to incorporate key influenza-related factors (e.g., vaccination rates, which directly modulate the size of susceptible populations, and viral antigenic variations, which affect transmission intensity) via a cross-modal feature fusion module to align these variables with frequency-domain epidemic signals.

## CRediT authorship contribution statement

**Tianyi Feng:** Writing – review & editing, Writing – original draft, Visualization, Software, Methodology. **Yu Huang:** Writing – original draft, Investigation, Formal analysis, Data curation. **Chunyan Luo:** Writing – review & editing, Supervision, Software, Investigation.

## Informed consent

Not applicable.

## Organ donation

Not applicable.

## Ethics statement

This article does not contain any studies with human participants or animals performed by any of the authors.

## Data availability statement

The data found in support of this study are available open source.

## Animal treatment

None.

## Generative AI

No generative AI or AI-assisted technologies were used in the writing of this manuscript.

## Funding

The authors declared that financial support was not received for this work and/or its publication.

## Declaration of competing interest

The authors declared that this work was conducted in the absence of any commercial or financial relationships that could be construed as a potential conflict of interest.
